# Citation network analysis for viewpoint plurality assessment of historical corpora: The case of the medieval rabbinic literature

**DOI:** 10.1371/journal.pone.0307115

**Published:** 2024-07-22

**Authors:** Nati Ben-Gigi, Maayan Zhitomirsky-Geffet, Binyamin Katzoff, Jonathan Schler

**Affiliations:** 1 Department of General History, Bar Ilan University, Ramat Gan, Israel; 2 Department of Information Science, Bar Ilan University, Ramat Gan, Israel; 3 Department of Talmud, Bar Ilan University, Ramat Gan, Israel; 4 Department of Computer Science, Holon Institute of Technology, Holon, Israel; Ariel University, ISRAEL

## Abstract

Citation networks enable analysis of author groups, defining in-group dynamics, and mapping out inter-group relationships. While intellectual diversity and inclusiveness is one of the important principles of modern scholarship, it is intriguing to explore the extent to which these principles apply to historical communities of leaders and intellectuals. This paper introduces a novel methodological framework aimed at assessing the degree of viewpoint plurality and diversity of historical scholarship communities, through an in-depth analysis of the citations used in their literature, which has become possible due to the recently developed advanced computational analysis techniques. To achieve this goal, we have devised a set of new network-based indicators grounded in standard network metrics. These indicators can be applied at both the individual author and community levels. The developed methodology was applied to a citation network automatically constructed from a corpus of Rabbinic Halachic literature spanning the 10th to 15th centuries. This corpus includes over 5,000 citations from hundreds of books authored by approximately 140 Rabbinic scholars from six diverse geographic communities. We found that most of the authors and communities cite many more external resources from other communities than their own reflecting a willingness to engage with a diverse range of viewpoints. A more in-depth analysis based on the novel proportional diversity measures unveils more intriguing insights. Contrary to expectations, communities with the greatest number of external citations, such as Spain and Ashkenaz, surprisingly exhibit lower levels of viewpoint plurality compared to others, such as Italy and North Africa, elucidating a key finding of the study.

## Introduction

In the past decades, citation analysis has been widely utilized in the fields of bibliometrics and scientometrics to gauge relationships and inter-influences between authors and their writings [1,2]. In addition, citation analysis is often used for revealing overarching trends within research fields that may impact critical decision making, such as industrial and economic growth priorities and allocation of funding resources [3].

In recent years, citation networks’ analysis introduces another layer, enabling examination of author groups, defining in-group dynamics, and mapping out inter-group relationships [4,5]. These relationships could span from neglecting a group to various forms of recognition, including dismissive, respectful yet differing, and full acceptance and agreement. When an author cites another’s works, even in disagreement, it implies that the cited author’s opinions hold weight or possess potential influence and authority. Such a presentation of multiple viewpoints naturally sparks debates among contributors, making it a fertile ground for research and understanding the relationships and openness levels among author groups. While intellectual diversity and inclusiveness are pivotal principles in modern scholarship, exploring the application of these principles to historical communities of leaders and intellectuals is a novel and underexplored research direction.

Therefore, this study introduces a novel methodological framework designed to assess the degree of viewpoint plurality and diversity within historical scholarship communities through a quantitative analysis of citations’ networks in their writings. This analysis has become feasible thanks to recent breakthroughs in computational text analysis techniques that enable automated citation extraction from historical corpora [6–8]. These corpora encompass texts written by a multitude of authors, spanning diverse time periods and geographic locations, thereby encapsulating distinct cultural paradigms and schools of thought. Therefore, they are fertile ground for research and understanding of the relationships between individual authors and entire communities, and their level of awareness of and openness to each other’s viewpoints.

The proposed methodology is based on a set of network-based indicators, incorporating standard metrics, such as degree centrality and new dedicated measures: 1) the external citations’ outdegree, and 2) resource diversity of an author or a group of authors. Unlike other methods for automated viewpoint detection and assessment in textual corpora [9], our research does not delve into the semantic processing of citations’ contexts, namely whether they challenge or reinforce a particular viewpoint. Instead, our emphasis lies on quantitative network-based evaluation, specifically highlighting the comparative volumes of external and internal citations for different authors, groups and communities.

To assess the feasibility and effectiveness of the proposed analytical framework, we automatically constructed a citation network of authors and books from a large corpus of Rabbinic Halachic literature from 10 to 15 CE, based on the multi-layered BERT-CRF [10] and BEREL Deep Neutral Network (DNN) models trained on the Rabbinic literature corpus [11]. The scrutinized corpus encompasses religious legislations and legal interpretations, featuring a plethora of citations. The constructed network comprises over 5,000 citations extracted and mapped from all the significant books written by approximately 140 Rabbinic authors, citing over 250 Rabbinic scholars in the following six main regions: North Africa, Spain, Provence, Italy and Balkan, France, and Ashkenaz (mostly modern-day Germany), which maintained intellectual ties during the abovementioned period [12].

The proposed network-based quantitative analysis delves into the patterns of intellectual exchange, enabling us to trace the multi-directional influences between various Rabbinic communities and address questions, such as the extent to which an author or a community incorporates content from its counterparts, the diversity of their referencing habits, and their overall level of viewpoint plurality.

Although the methodology was applied to Rabbinic literature, it can be easily adapted to any multi-viewpoint historical corpus. The code developed for this analysis is available on GitHub at https://github.com/NatiBenGigi/plurality_in_the_medieval_rabbinic_literature.

## Related work

The incorporation of an inclusive and balanced representation of multiple viewpoints stands as a fundamental tenet in the contemporary scientific approach across various disciplines [13]. Building on the work of [14,15], assert that "scientific theories which are the result of scientific discoveries are not immutable facts that are true at all times but can be overturned by other competing theories" (p. 190).

Automatic identification and assessment of diverse viewpoints within textual corpora can be achieved through the examination of linguistic features, grounded in the hypothesis that similar viewpoints generally manifest in similar language usage. Linguistic features explored for their predictive value include word frequency representations (bag of words), word embeddings, named entities, part of speech (POS) analysis, dependency structures, sentiment analysis, and domain lexicons [16]. These features have been implemented in both supervised and unsupervised machine learning and deep learning algorithms. However, these algorithms exhibit limitations in their effectiveness when applied to historical texts, especially in national languages, such as Hebrew, that are the focus of this research [17]. An alternative approach for scientific and literary data exploration widely employed in bibliometrics and scientometrics is citation network analysis which delves into citation patterns to identify pivotal articles, authors, and journals [18]. This approach does not require a deep semantic analysis of the entire text of the corpus, but only of the citations that appear in it. Therefore, in this study, we propose the utilization of network-based indicators to assess the plurality of viewpoints in historical corpora. The subsequent subsections delve into a review of related work in the field of citation analysis, focusing on both contemporary and historical corpora.

### Citation network analysis in contemporary scientific corpora

Citation network analysis stands as a prominent tool in science mapping, relying on large databases of scientific documents [19,20]. Its goal is to reveal aggregated relationships among publications and authors, offering insights into topics, sub-fields, communities, emerging trends within a given discipline, and their interconnections. Additionally, it can provide global maps of cross-disciplinary relationships for multi-disciplinary bibliometric corpora [21–25]. However, using citation metrics to understand the diversity of communities or individual nodes in a network also has drawbacks. These include potential biases in citation practices, oversimplification of network dynamics, temporal lags in data, and a focus on the quantity of citations rather than the quality of the research [22,26].

Within the scientific world, the examination of relationships and knowledge impact between communities, along with its effects on these communities, has been investigated from various perspectives. McLevey et al. [27] devised statistical network models to examine the impact of philosophers of science publishing in and being cited by science journals on philosophers’ standings within their citation networks. They employed Exponential Random Graph Models [28] considering factors such as connections, popularity (geometrically weighted indegree), activity (outdegree), clustering (geometrically weighted edgewise shared partners), and interconnected paths (geometrically weighted dyadwise shared partners). The model was later expanded to include additional variables such as publication venue, author count, and others. Their findings indicated that philosophers of science frequently publish outside their field, and their work is often cited in scientific journals. However, these cross-disciplinary interactions do not affect their influence within their own academic circles. Generally, philosophers seem unaffected by works published in external journals, and those cited by non-philosophers do not experience an increase in citations from their philosophical peers.

Gates et al. [29] leveraged citation analysis, using citation and reference data from the Web of Science, to assess the interdisciplinarity of scientific research. They categorized articles into disciplines, analyzed co-citation networks, and used quantitative metrics like the Rao-Stirling diversity index [30] to measure the diversity and interconnectivity of disciplines across scientific articles. Their findings showed an increase in interdisciplinary citations and references over time, indicating that scientific research is becoming more integrated across different fields. This trend underscores the importance of collaborative approaches in addressing complex challenges. Another aspect of knowledge diffusion was researched by Zhu et al. [31], who viewed it as a trading process between disciplines. They examined this within Computer Science subfields using a 15-year citation dataset, highlighting the significant influences of subfields such as Computer Science Applications and Artificial Intelligence. The study employs various techniques, including assessing incoming citations, cited/citing ratios, and the diversity of citing areas to gauge influence and interdisciplinary impact. Network Analysis Metrics like PageRank [32] and betweenness centrality [33] measure the importance and connectivity of subfields within the citation network. The research identifies robust trading relationships among subfields, signifying strong networks that enhance the exchange and impact of knowledge, akin to economic trade.

As part of the research on connectivity and influence between communities, the importance of the betweenness centrality measurement in network analysis was highlighted by the work of [34]. They investigated the application of network centrality metrics—closeness, betweenness, and eigenvector—to identify and assess the significance of research papers within a journal, using a dataset from the Public Library of Science (PLOS) to construct a co-citation network. In this context, they found that betweenness centrality is highly effective in multidisciplinary journals by pinpointing papers that serve as informational bridges within and between diverse research communities. This bridging role facilitates inter-community knowledge transfer, making betweenness centrality a critical tool for researchers navigating the interconnected spaces of academic disciplines.

While numerous studies have explored citation network analysis across various contemporary scientific disciplines over the past decades [23,35,36], its application in the humanities and historical corpora has been relatively limited [37–39]. Additionally, those studies have a predominant focus on several key areas: (i) Comparisons of the humanities to other disciplines in terms of growth timeframes, internationality, and interconnectivity [37]; (ii) exploration of the potentially problematic role of bibliometrics in performance evaluation within the humanities [40,41]; (iii) mapping the cognitive and social structure of the humanities, such as understanding the relationships among various disciplines and sub-disciplines in institutional frameworks and personal relations [19,42,43]. Challenges in this field include diverse publication practices in the humanities and the absence of comprehensive data sources and tools like the SCI (Science Citation Index) and SSCI (Social Science Citation Index) [37–39,44–46]. Must [47] suggests several added values that humanities citation databases and their large-scale analysis could provide, including sustainability and re-actualization of past studies, tracing the formation of schools and international cooperation communities, facilitating the connection and reuse of sources traditionally accessible to and studied by a limited number of individuals, and exposing a variety of sources to large communities of scholars.

### Network analysis of historical corpora

Network analysis has been performed on historical texts, though such networks have more often been constructed based on social links and interactions (e.g., letter senders and receivers) rather than citations [48–53]. Murai et al. [54] presented an automated technique for quantitatively representing thoughts of different authors in a network by analyzing the clusters produced in the clustering process of the network. Utilizing this technique, the study constructs and examines the co-citation networks of five prominent Christian theologians throughout the history. It differentiates them based on their respective sects and historical periods, complementing traditional literary approaches in the fields of philosophy and theology. Bar-Asher Siegal and Yovel [55] employ network analysis to challenge the notion of a distinct separation between early Judaism and Christianity. Their research underscores the dynamic interplay between different religious traditions, revealing significant mutual influences. Recently, the evolution of intellectual communities has been analyzed using network analysis by Petz et al. [56]. This study harnesses a unique Linked Open dataset and a community detection algorithm to track and understand the formation, growth, and structural patterns of these communities over time.

Several studies analyzed citation networks of modern scholars citing historical authors and books (primary sources). Thus, Romanello [6] explores how canonical citations in ancient classical texts can be utilized to study intertextuality. He demonstrates the use of natural language processing techniques for extracting citations and identifying entities in the text, treating citations as a unique type of entity for extraction through Named Entity Recognition methods. For this purpose, a dataset was created from a selection of reviews in L’Année Philologique dating back to 1924, aimed at training an algorithm to autonomously extract citations and assess the accuracy of this extraction process. Colavizza [7] investigates a citation network among recent monographs on Venetian history. The network comprises monographs as nodes and their citations as links. Within this network, it is possible to discern clusters of monographs by specific disciplines (e.g., architecture history and art history) or time periods (e.g., the Middle Ages and the early modern era). This network-based mapping offers an overview of current trends in Venetian historical studies and highlights a group of extensively cited works central to the scholars in the field.

Blidstein and Zhitomirsky-Geffet [45] present a novel generic framework for generating and analyzing citation networks in historical humanities, distinguishing between primary sources (historical texts) and secondary literature (modern scholarship). This approach is tailored to humanities’ unique citation practices and involves variables like source and citation types. Applied to a large corpus on ancient Mediterranean religions, it enabled constructing and analyzing diverse networks. These analyses revealed distinct structural patterns in the discipline, underscoring the value of integrating multiple network types for a comprehensive understanding of humanities research.

### Analysis of historical networks in Rabbinic literature

Several studies attempted to construct and analyze citation networks from Rabbinic texts. HaCohen-Kerner et al. [57] developed a system to identify citations in modern Rabbinic Responsa texts written in the last 130 years using a supervised machine learning model with over 100 features including orthographic, quantitative, keywords, and n-gram models. They primarily addressed cases with single citations, finding logistic regression to be the most effective. Koppel and Schweitzer [58] leveraged HaCohen-Kerner et al.’s [57] system for the analysis of citations to determine influence networks in Jewish Responsa literature, tracing both direct and indirect influences.

Zhitomirsky-Geffet and Prebor [59] employed a semi-automatic technique to generate SageBook, a network mapping the relationships of Jewish sages across generations within Halachic debates in the Rabbinic literature. Their approach hinged on lexical and syntactic patterns indicative of the sages’ names and their relationships (e.g., agreement, citation, or a complementary view). This methodology was successfully tested and validated on the Mishna corpus. Zadok et al. [60] extended this study by constructing and analyzing the networks of the Jewish sages in the Mishnah and Tosefta, Jewish canonical sources dealing with civil and religious law redacted in the third century CE, as noted by Rosen-Zvi [61] and Mandel [62], to discern whether these historical literary works were edited by the same person(s). This was done by comparing editorial style profiles of these works based on standard metrics of their networks. The method, focusing solely on network features rather than linguistic stylometric analysis, revealed distinct network-based profiles indicating different editorial styles, despite substantial thematic and linguistic similarities of the two texts.

Waxman [53] introduced a comprehensive social network for the Babylonian Talmud that spans multiple generations. This database visually maps out mentorship and local interactions between Rabbis, who co-occur in the Talmud debates. The process involved identifying the names of sages in the Talmud texts from the Sefaria database (https://www.sefaria.org.il) in Hebrew and their English transliterations, highlighting key connections. Similarly, Satlow and Sperling [63] utilized quantitative social network analysis techniques to construct and study the citation network within the Babylonian Talmud. Their goal was to uncover insights about the historical connections among Rabbis and the transmission pathways of the Talmud’s sources. Their research revealed that the Rabbinic network in the Babylonian Talmud consists of various unique communities, all connected by a dense core network formed through the relationships of the most influential Rabbis. Prebor et al. [64] used machine learning algorithms to predict the missing origins of Medieval Hebrew manuscripts based on the other existing metadata and, through this, create an evolving network that traces the history, movement, and exchange of ideas and content within Jewish culture across various regions. In the process, their work also reveals migration trends within Jewish communities reflected in the manuscripts’ metadata.

While the reviewed above works utilized citation and social network analysis of historical corpora for various tasks and goals, to the best of our knowledge, this is a first attempt to employ citation networks to assess the viewpoint plurality and diversity of historical texts.

### Traditional biographical research on intellectual communities of Jewish sages from the Middle Ages

Extensive research conducted on Jewish sages active during the first half of the second millennium, known as the "Rishonim", delves into the mutual acquaintance and influence among Jewish intellectual communities across various geographic locations during the Medieval period. This scholarship, as evidenced by the works of Grossman [65,66], Ta-Shma [12,67], Galinsky [68], Emanuel [69], Kanarfogel [70], Reiner and Roth [71], Reiner [72], and Roth [73] and others, places significant emphasis on exploring literary familiarity, citations, and legal influence among these communities within the context of Jewish law and exegesis.

For example, Kannerfugel [70] studied the familiarity with the writings of sages from Ashkenaz and France, known as the “Tosafists”, among Jewish scholars in Spain during the High Middle Ages. His research describes the relatively limited utilization of their literary works in the twelfth century, followed by a gradual rise in usage throughout the thirteenth century, culminating in substantial engagement by the Ritva (Rabbi Yom Tov b. Abraham al-Ishvilli) around the transition from the thirteenth to the fourteenth centuries. Similarly, Ta-Shma [12] described the influence of the Rambam, active in twelfth-century Egypt, on scholars from Italy by scrutinizing citations from his works within their writings. Likewise, Galinsky [68] investigated the dissemination process and the magnitude of influence exerted by the work of “Arba Torim” (Rabbi Jacob Ben Asher), penned in Spain during the first half of the fourteenth century, by tracking quotations from the book by other authors.

These studies, along with others of their kind, elucidate significant phenomena by tracing citations, shedding light on the connections between the specific sages under examination. However, a comprehensive approach leveraging large-scale computational citation analysis, coupled with quantitative network analysis techniques, to determine the degree of viewpoint diversity within a vast Rabbinic Halachic corpus, has not yet been introduced.

## Methodology

### Citation network construction

Our approach to determining the viewpoint plurality level of an historical corpus is based on citation network’s indicators. Thus, the two initial steps for applying this approach require the citation network and its metadata ontology construction for the corpus under study:

1. Citation Network Construction: A specially designed system is employed for the task of citation extraction from text, which accepts the complete corpus as input and returns a comprehensive list of all identified citations. It is essential to adapt this system to the specific nature of each corpus. These citations form the network, where nodes represent authors (and/or their books) and edges signify citations among them.
2. Metadata Ontology Construction: The second step consists of creating an ontology that includes complementary information regarding the resources under analysis and their authors, as well as the authors and books referred to in the extracted citations. This data is utilized in grouping the citations in the constructed network into communities based on common ontological properties of authors and/or their works, such as geographic locations, time frames, and other characteristics.

### The network-based indicators of the viewpoint plurality

Following the construction of the network and its communities, the next step focuses on the analysis of the network’s data based on two new dedicated indicators that reflect the viewpoint plurality of the corpus. The devised indicators are based on the widely used outdegree centrality metric [74], adapting and refining it to quantify the individuals’ and communities’ viewpoint diversity and inclusion.

1. Resource diversity: the number of distinct external resources from other communities in the network out of all the distinct cited resources;
2. External citations’ outdegree: the number of outgoing citations of other network communities’ resources out of all the outgoing citations.

The resource diversity represents the awareness and inclusiveness of varying viewpoints, while the external outdegree metric captures the extent of the plurality and utilization of different viewpoints. We implement these two indicators at two levels: 1) for individual authors, and 2) for entire communities. For an individual author, the first indicator is determined by counting the number of distinct external authors (i.e. authors from other communities) cited by a given author out of all the distinct authors (external and internal) cited by this author. The second indicator is based on the total number of outgoing external citations made by an author out of all the outgoing (external and internal) citations of this author. At the community level, we calculate the percentage of distinct external authors cited by the community as a whole out of all the distinct authors (external and internal) cited by the community. The second indicator is a percentage of external outgoing citations of all the authors in a given community out of all the outgoing citations of this community, when each citation between the same pair of authors is counted only once.

**Fig 1:**
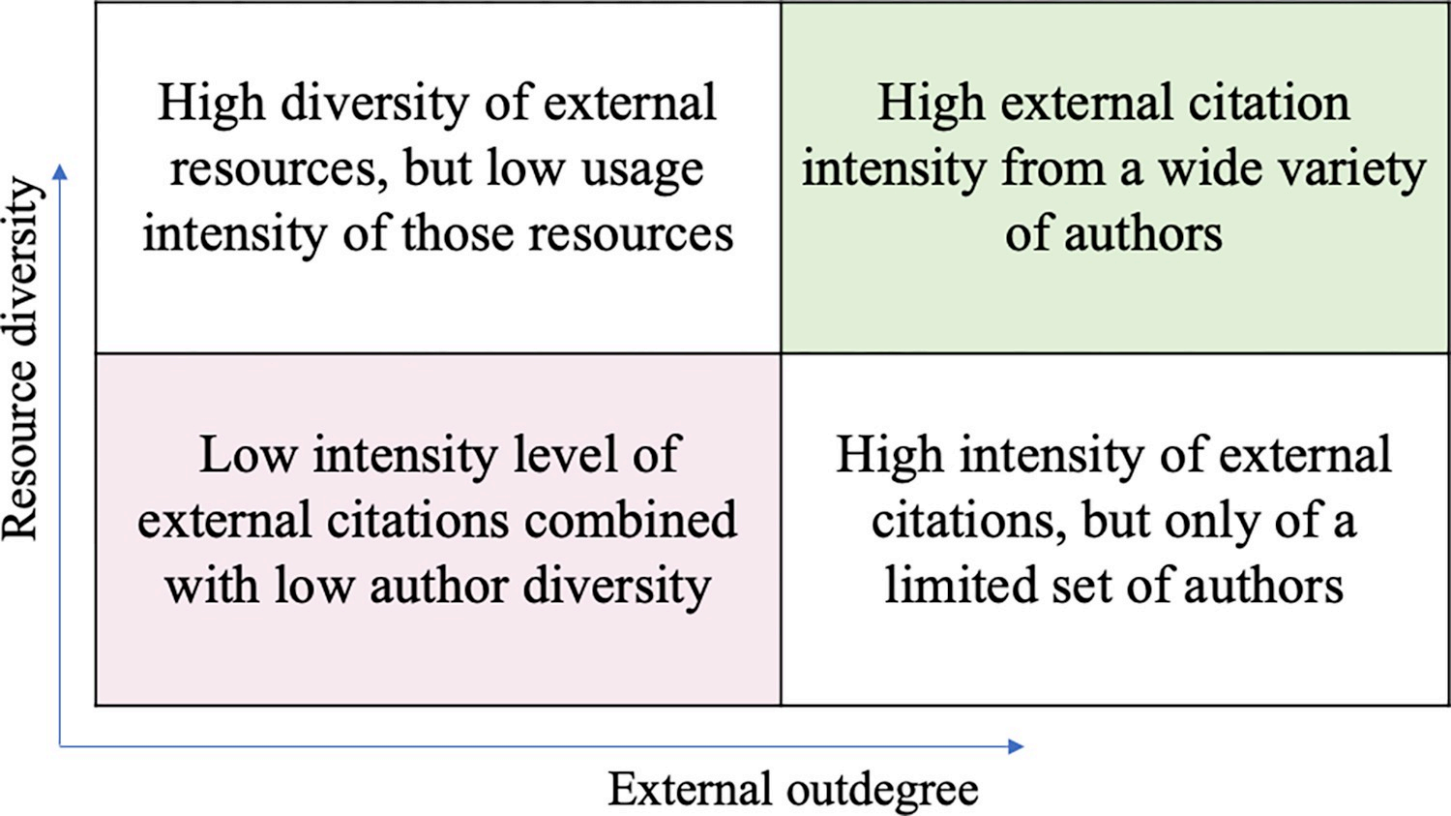
A bi-dimensional chart plots external degree (X axis) against resource diversity (Y axis).

To visualize the overall plurality of the different authors and communities based on the above two indicators, we use a bi-dimensional representation as shown in Fig 1.

A bi-dimensional visualization with external degree on the X axis and resource diversity on the Y axis, divided into four quadrants indicating varying levels of plurality.

In addition, our analysis uses standard network metrics, such as the network connectivity and average degree centrality as well as descriptive statistics of the corpus, such as the number of citations per author and community and the number of books and authors in each community. We note that these indicators do not explain the reasons for a group’s viewpoint plurality level. The reasons for the detected phenomena could be explored by domain experts, while our methodology remains domain agnostic, making it suitable for any sufficiently inclusive and comprehensive corpus.

### Experimental setting: The use case of the Rabbinic Halachic literature

We implemented our methodology on the Rabbinic Halachic literature dating from 10 to 15 CE. This corpus consists of hundreds of books penned by around 120 authors, encompassing the bulk of the Halachic literature of this era, including all significant writings. This corpus is notable for its combination of legal directives and interpretative content, both of which naturally involve numerous citations. The variety of authors of the corpus further enriches its depth. While all were of Jewish descent, their backgrounds varied due to their residence in either Christian or Muslim countries. The diversity in origin has introduced nuanced variations in behavioral and intellectual norms, which are evident in the corpus texts. This has resulted in the formation of communities or schools of authors, where each community consists of individuals sharing similar approaches to teaching and legislation. For instance, as demonstrated by Grossman [75], there are substantial cultural impacts on Halakhic laws concerning women specifically stemming from the distinctions between Christian and Muslim countries. Representing the six main communities of the corpus [12], such as North Africa, Spain, Provence, Italy, France, and Ashkenaz (mostly modern-day Germany), the corpus is abundant in citations and contains discussions of an interpretive or codexical nature, as well as including Responsa literature as part of its composition. It incorporates citations to approximately 200 Rabbis and 250 books, along with copious citations of the Babylonian Talmud and other resources. However, in this study, we exclusively examine authors (or their books) from the specified historical period, deliberately omitting citations of earlier works. The final corpus of the study contains a total of 62.5 million tokens.

It should be noted that in some communities, there was no continuous Jewish settlement producing Rabbinic Literature during the investigated period. For example, in the 14th and 15th centuries, there is a lack of Halachic literature in France due to the persecution of Jews in the country [76]. Similarly, in North Africa during the 13th century and parts of the 14th century, Jewish scholarly output was significantly disrupted. The lack of continuity in Rabbinical writing in some places poses a significant challenge for those who wish to describe the degree of relative circulation and influence of compositions from different places. However, the focus of the current research is the description of the variety of sources that were used in the literature created in the examined period, which is feasible despite the lack of continuity. Future research investigating the internal dynamics of these communities will need to account for these constraints.

The geographic division into scholarly communities presented above is the accepted division in the scientific research literature concerning the intellectual centres of the Jews during the period under study [12,67]. Additional distinctions, like the separation between Ashkenaz and France as explored in Grossman’s studies [65,66], or the intricate relationship between Provence and France as discussed in Roth [77], further delineate these division. However, this classification suffers from certain inaccuracies. For example, the category "North Africa" includes eastern regions like Tunisia and Egypt, where scholars, such as Rabbi Hananel and Maimonides, were active, as well as western countries like Morocco and Algeria, where sages like the Rif and the Duran family worked [12,67]. These regions are much farther apart from one another than most of the communities in Europe, which are considered distinct, and yet are included under the same community in this classification. Additionally, Jewish literature written in Arabic in more eastern regions, such as Baghdad, is not included in this categorization due to its relatively small volume and unique characteristics. This body of work should be further explored in more detail as part of a follow-up study. Even though there can be movement within these borders by sub-communities, in classical research literature in Jewish, the borders of the High Middle Ages are relatively fixed. For example, in Spain, the Reconquista caused significant internal shifts within Jewish communities, leading to migrations between the northern and southern regions of the country [76]. Despite these movements, the Jewish population in Spain is generally considered as a single, unified community [78]. However, when examining the differences among various Batei Midrash (academies) within Spain, these distinctions are carefully analyzed.

In our previous study (Ben-Gigi et al. [Unpublished]), we developed a dedicated citation extraction system that was applied on the Rabbinic literature corpus described above. The system consists of the following components:

1. Two DNN (deep neural networks) models: These models, based on the Bidirectional Encoder Representations from Transformers—Conditional Random Field (BERT-CRF) architecture [10], were tailored and trained specifically for Rabbinic literature. They carry out two distinct tasks: identifying the boundaries of a citation within the text and recognizing the essential components of the citation within those boundaries. The results for both tasks demonstrated quite good quality. For the first task, the precision was 0.909, the recall was 0.884, and the F1 score was 0.896. For the second task, the precision was 0.845, the recall was 0.867, and the F1 score was 0.856.
2. Name normalization: After identifying the citation elements, the system allocates standardized names to each component based on a predefined synonym table. This table was pre-developed through a semi-automated process, where names of authors and books were extracted automatically and then manually linked to their corresponding canonical names.
3. Citation validation and completion: The final step involves validating the compatibility of the citation components and attempting to complete missing parts using the ontology detailed below. The ontology contains details about the authors, including information about their books and biographical data [65,79].

By integrating the two DNN models for citation extraction with name normalization and citation completion, we achieved final results with a precision of 0.896, a recall of 0.905, and an F1 score of 0.901.

After the citation extraction phase, we represent the obtained citation data and metadata as an ontology based on CIDOC-CRM [80] (see https://www.cidoc-crm.org for further information), a living standard (ISO 21127:2014) for cultural heritage knowledge representation. CIDOC-CRM is designed as “a common language for domain experts” and “allows for the integration of data from multiple sources in a software- and schema-agnostic fashion” [81]. It has been applied as a base model and extended in many domains related to cultural heritage. In this study, it was chosen as the basis for defining the citation domain ontology due to its standard and generic nature. This enables n-ary, rather than binary, relationships between entities in the ontology, as required for representing bibliographic and biographic data. The constructed ontology contains instances of two explicit classes: crm:E21_Person and crm:E84_Information_Carrier, which are linked by crm:P67_Refers_to. This represents books referring to other books or authors. Additionally, several implicit classes are used: crm:E52_Time_Span, crm:E53_Place, crm:E67_Birth, crm:E69_Death, and crm:E78_Collection.

**Fig 2:**
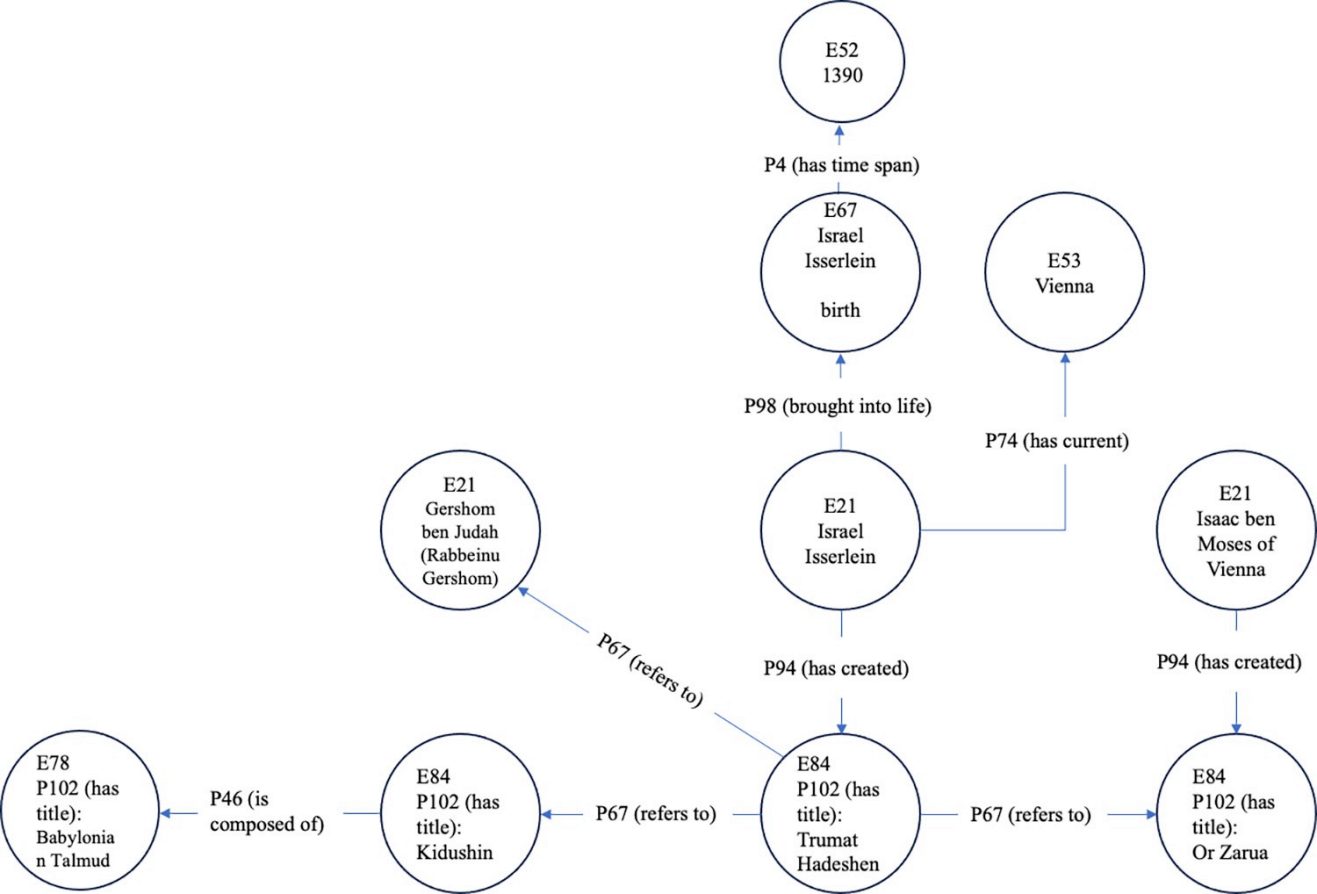
Sample CIDOC-CRM-based knowledge graph of books, authors, and citations of the medieval Rabbinic corpus.

Fig 2 illustrates a fragment of the obtained ontology for cross-reference representation of books and authors. The primary book, "Trumat Hadeshen," is an instance of the Information Carrier class (E84), authored by Israel Isserlein, who is represented as an instance of Person (E21). This book cites other authors, notably Gershom ben Judah (Rabbeinu Gershom) (E84 via P67 to E21), and other books like "Or Zarua" (authored by Isaac ben Moses of Vienna (E84 via P94 to E21)) and the "Kidushin" tractate of the Babylonian Talmud (E84 via P67 to E84, and E84 via P67 to E84 to P46 to E78, respectively). The graph also contains information about the author Israel Isserlein, including the event of his birth in 1390, represented by an instance of E67 linked with E52 (E21 via P98 to E67 via P4 to E52), and highlights Vienna as the primary place of his activities (E21 via P74 to E53).

## Results

The obtained citation network comprises 206 nodes (citing and cited Rabbis) 5050 4110 edges that represent citations between authors (with 4110 distinct citations). Table 1 provides descriptive statistics of our citation network data and metadata. For each community in the corpus it displays the total word count (tokens) in its books, the overall number of Rabbis (Encompassing those who did not write books included in our corpus, but were cited by books from our corpus, it is noted that during the examined period, students often moved to study with various Rabbi-teachers, sometimes for several years. However, not all of these teachers and students authored books; sometimes, they are only referred to in books written by their peers or students [65]. The number of authors in the community who wrote some books, the quantity of books authored by them, and the aggregate number of citations (both internal and external) in the community’s books. Furthermore, the table shows each community’s average outdegree centrality, the text citation rate representing the percentage of citation tokens out of the total token count, and the average number of citations per author within each community. The figures in the table indicate that Spain and Ashkenaz are the two largest communities in the corpus with top numbers for all the parameters related to the community size (the parameters that appear in the five left columns of the table). In contrast, Italy and North Africa have the lowest number of authors, with Italy having a much lower token number than North Africa. Yet, Italy exceeds both North Africa and even France in the number of citations, despite its lower token count and has a second-top author citation rate after Ashkenaz. Furthermore, while the token counts for Ashkenaz and France are roughly similar, France achieves this with less than half the number of authors compared to Ashkenaz. On the other hand, the number of citations from Ashkenaz is three times higher than that of France. France, on the other hand, scores the lowest in text and author citation rate indicators.

**Table 1:** Descriptive statistics for each community.

Community	Ashkenaz	France	Italy	N. Africa	Provence	Spain	Total/Avg
**Tokens no.**	8,834,131	8,752,909	4,806,764	7,706,199	7,352,181	25,248,699	62,700,883
**Rabbis cited**	53	34	13	17	29	60	206
**Authors**	32	14	11	9	15	43	124
**Books**	51	30	17	26	29	97	250
**Distinct citations**	1202	367	379	361	401	1400	4110
**Distinct external citations**	783	228	346	311	324	920	2912
**Avg out degree**	0.34	0.21	0.38	0.33	0.29	0.23	0.18
**Text citation rate (%)**	0.41	0.11	0.25	0.18	0.13	0.34	0.37
**Author citation rate**	44.51	17.46	37.9	32.81	25.06	31.11	33.14

**Fig 3:**
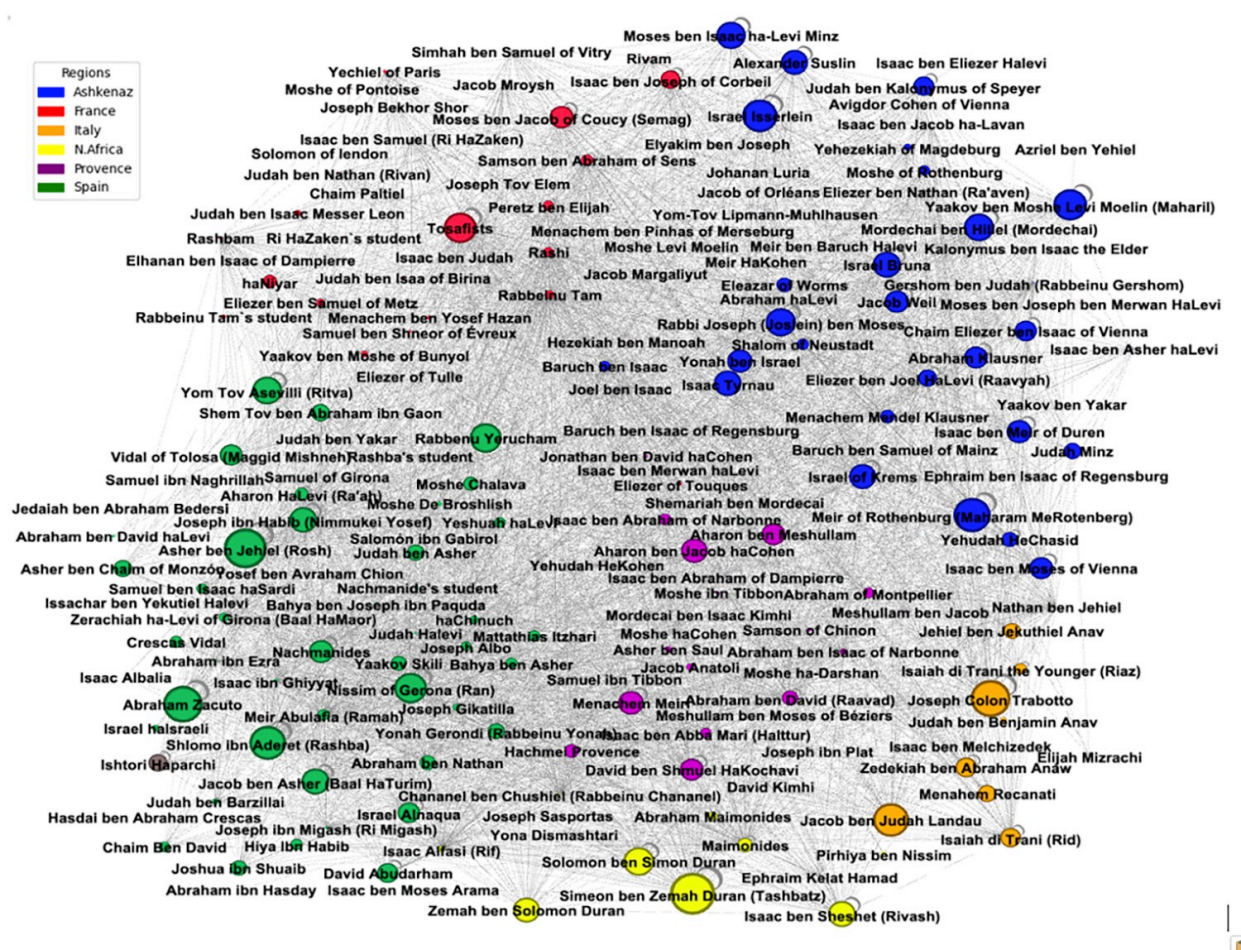
The entire corpus citation network diagram.

The average degree centrality of our network stands at 0.22, indicating a robust level of interaction and exchange of information. This dynamism is somewhat reflected in the outdegree centrality, recorded at 0.18, suggesting a substantial authors’ engagement in citing others. However, the indegree centrality at 0.12 reveals an uneven pattern of network activity. This suggests that while certain authors are frequently cited, a larger number of authors are cited less often, thereby reducing the overall average of indegree centrality. In Fig 3, the network graph clearly highlights Ashkenaz (blue nodes) and Spain (green nodes) for their leading number of outgoing citations relative to other communities. Moreover, the proportion of authors with a high outdegree centrality varies across communities. In Spain and Ashkenaz, a significant number of authors have high outdegree citations. In contrast, in the French community, which has a medium-sized author pool, only three authors are prominent in terms of outgoing citations.

Authors are represented as nodes and citations are represented as edges. Created using Gephi, the size of each node is determined by the represented author’s outdegree centrality, which is the number of the author’s citations of other authors in the networks. The community definitions adhere to the traditional research methodologies found in the Jewish studies research literature as explained above.

**Fig 4:**
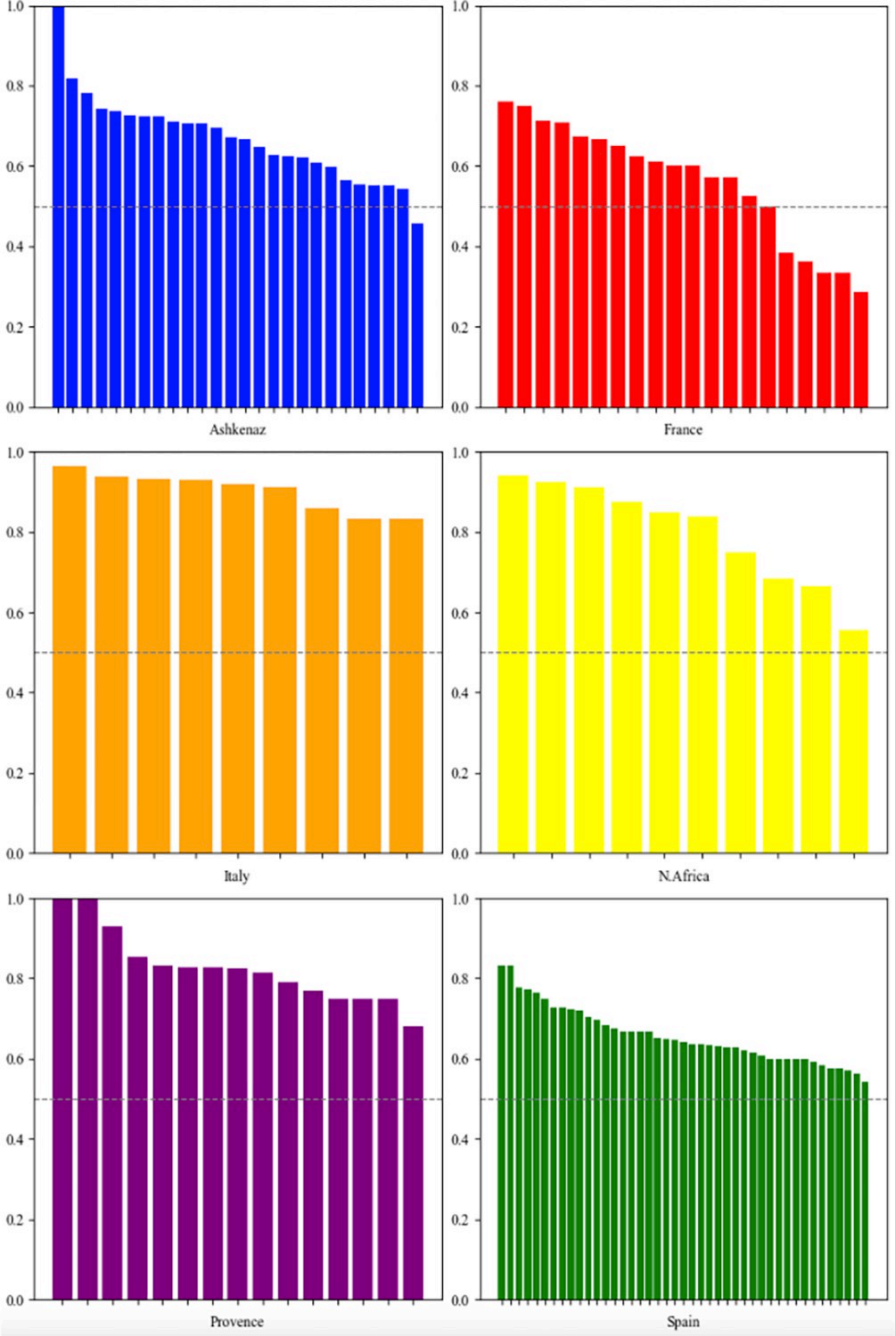
Resource diversity for each author across communities.

First, we apply our indicators at the individual author level. Fig 4 illustrates the extent of resource diversity by each author in every community. Authors with a resource diversity usage score under 0.5 possess a resource set that is predominantly composed of internal rather than external authors. Notably, in France, approximately one-third of the authors demonstrate a relatively low resource diversity level, below 0.5. Yet, the observation that most authors have a score exceeding 0.5 suggests that a significant portion of their resources are external. Overall, authors from Ashkenaz, Spain, and France generally exhibit a lower level of resource diversity compared to those in the other three communities. Conversely, Italy shows the highest level of resource diversity for each author. One author in Ashkenaz and two in Provence (Yehezekiah of Magdeburg, Jonathan ben David HaCohen of Lunel, and Samson of Chinon) display a resource diversity level of 1. This implies that they do not cite any resources from their own community at all. This finding warrants further investigation in future research.

A diversity level above 0.5 signifies that the majority of cited resources in an author’s resource set are external.

**Fig 5:**
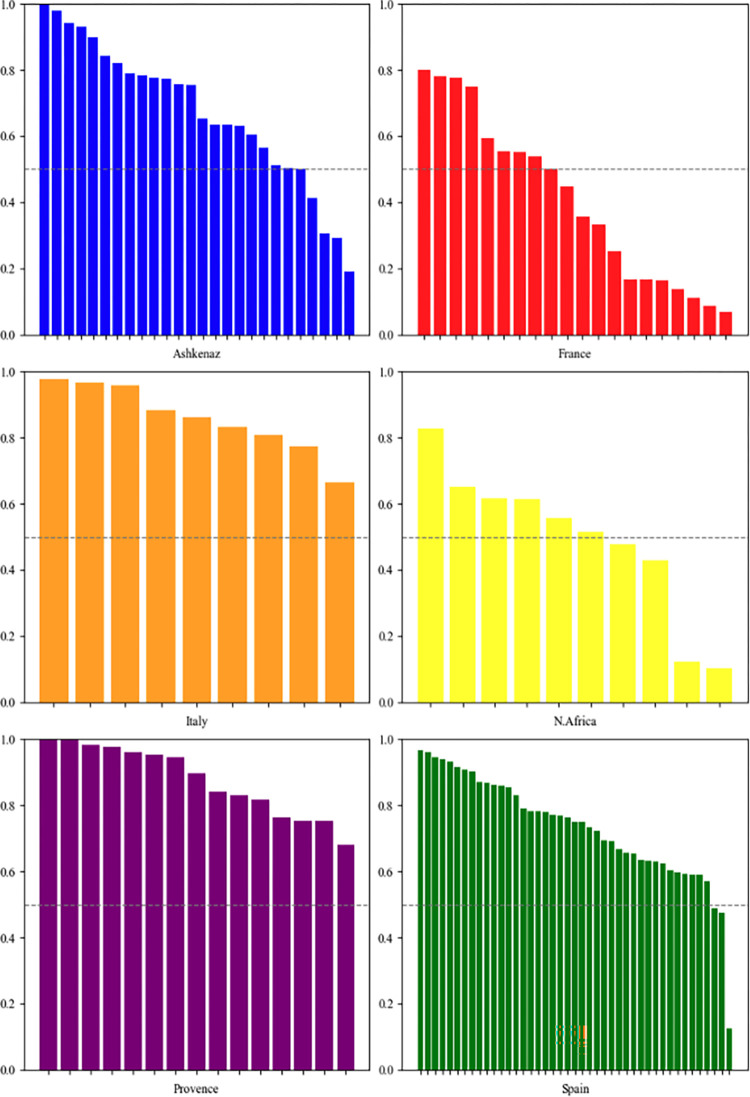
External outdegree for each author across all the communities.

Fig 5 depicts the external citations’ outdegree values of every author in different communities. Similar to Fig 4, a value exceeding 0.5 suggests that an author mainly cites external authors. Generally, for four out of six communities (Ashkenaz, France, Italy and North Africa), the scores of this indicator are lower than those of the resource diversity indicator. This implies that while authors tend to mostly cite various external resources, they cite internal resources from their own communities more intensively. Still, as most authors’ scores surpass 0.5, it indicates an overall tendency to cite more external resources than internal ones. The notable exception is the French community, where most authors predominantly cite peers within their own community. In contrast, in Spain, Italy and Provence, a significant majority of authors show a strong inclination to cite external resources over internal ones.

A score exceeding 0.5 indicates that the majority of the authors’ citations are to authors outside their own community.

**Fig 6:**
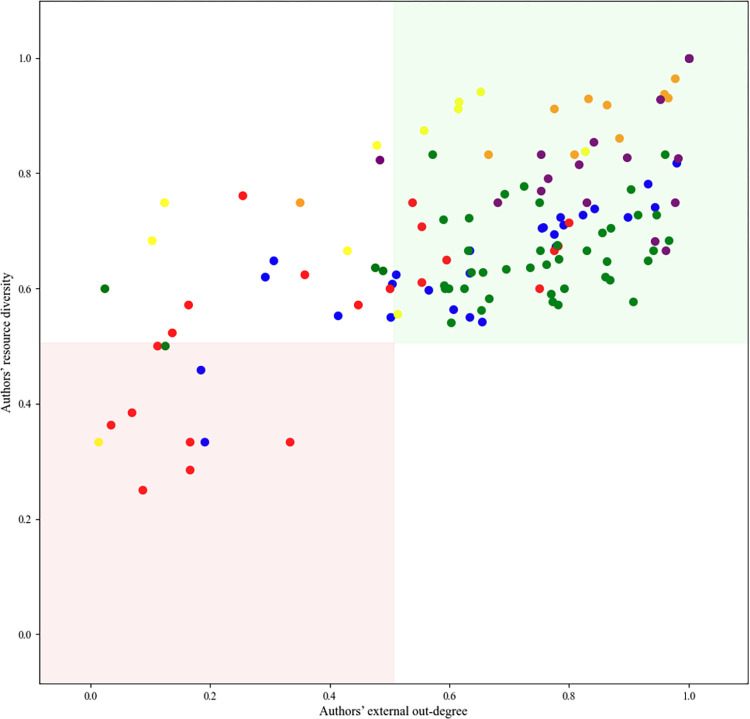
A bi-dimensional visualization illustrates the final plurality level for each author.

Fig 6 displays the combined plurality level incorporating both resource diversity and external outdegree for each author on a bi-dimensional graph. Most authors are positioned in the top right square, signaling a high score in both metrics. Upon closer examination, it is observed that authors from Provence and Italy predominantly occupy the upper section of the top right square, whereas those from Ashkenaz and Spain are more often found in the lower part of this square. North African authors are distributed across the top two squares, indicating that they all cite a wide range of external resources but to a limited extent. Authors from France are scattered across the top squares and the bottom left one, suggesting that there is no single characteristic applicable to all French authors. A similar observation can be made for Ashkenaz authors, albeit more subtly, as most authors from this community are positioned within the top right square. Finally, it should be noted that there are no authors positioned in the bottom right square. This indicates an absence of authors who intensively cite only a limited set of external authors.

This visualization maps resource diversity on the Y-axis and external citations’ outdegree on the X-axis. Each author is assigned a color based on their community of origin: Ashkenaz is blue, France is red, Spain is green, North Africa is yellow, Provence is purple, and Italy is orange.

**Fig 7:**
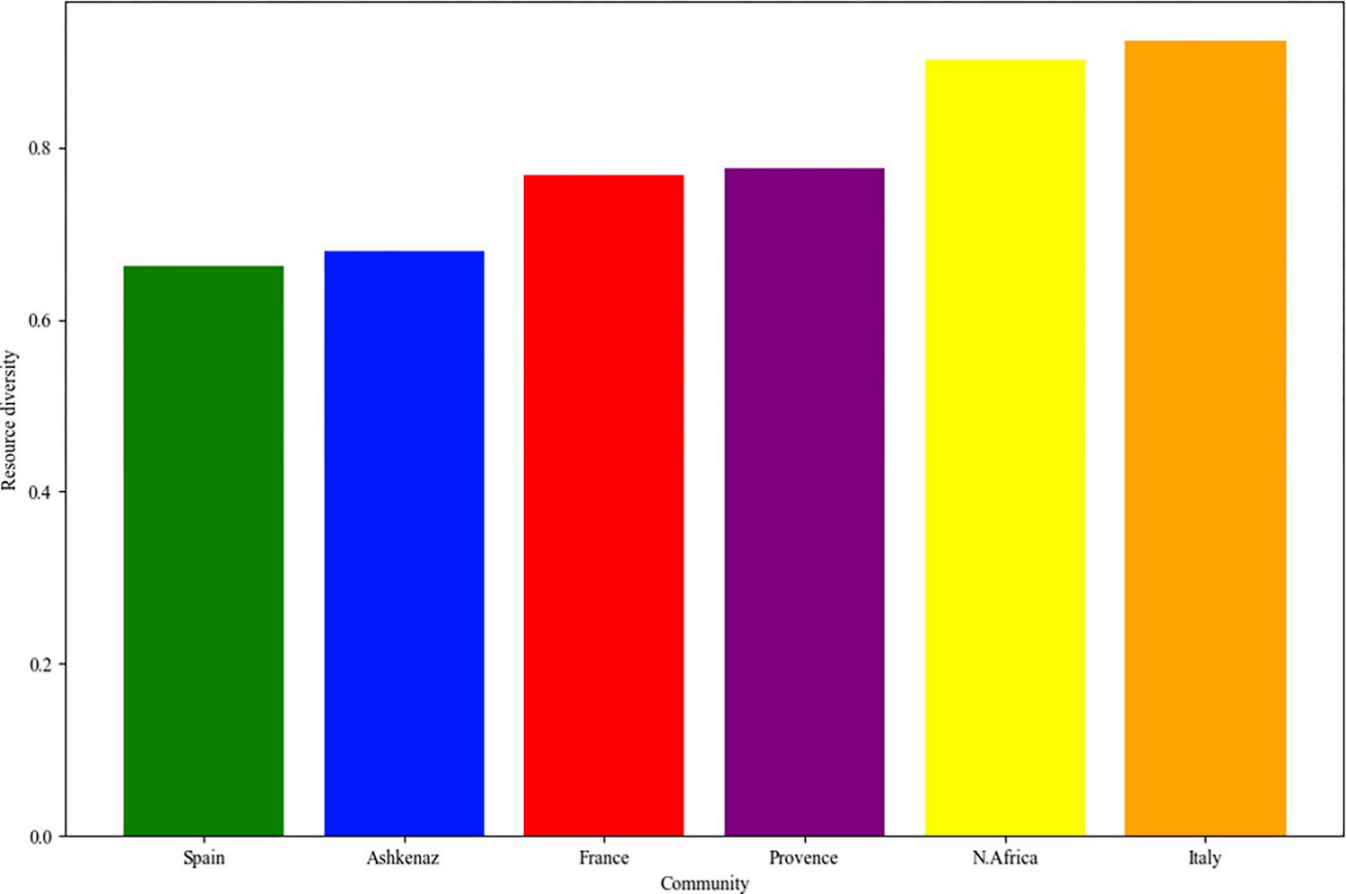
The resource diversity indicator as a percentage of unique external authors cited by each community.

When employing the first indicator (resource diversity) at the community level, it can be observed that Italy exhibits the top scores, whereas Spain presents the lowest scores (see Fig 7).

**Fig 8:**
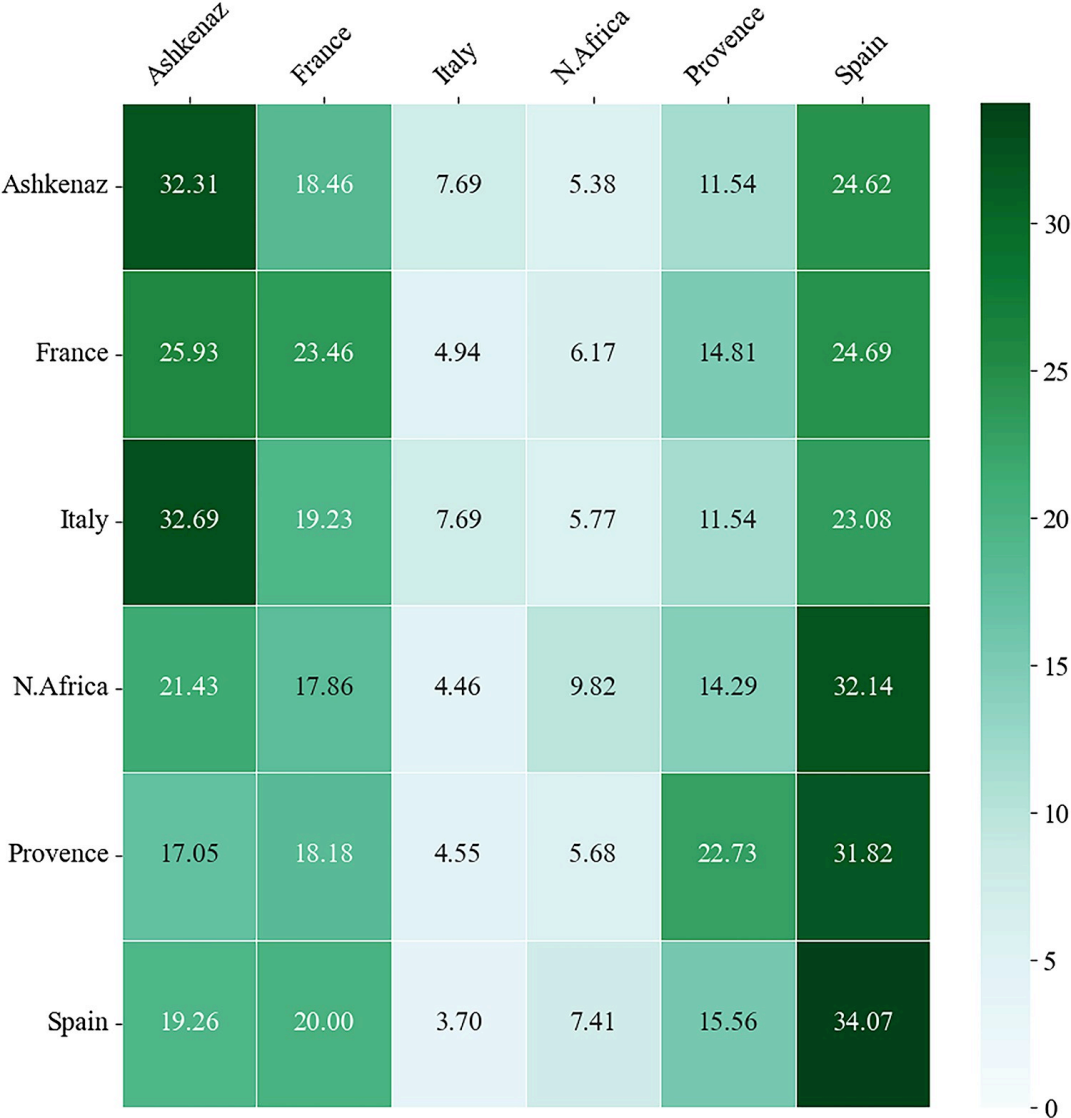
Cross-community resource diversity heatmap.

When examining the heatmap in Fig 8 that illustrates the resource diversity cited by each community, it is apparent that the greatest levels of self-citation of distinct authors appear in Spanish and Ashkenazi communities (32–34% of all distinct cited authors), the two largest communities in the corpus. Italian community massively cites diverse authors from Ashkenaz (33%), while North African and Provence communities cite many different Spanish authors (32%). In France, the pattern of citations is more balanced in terms of resource diversity, with a nearly equal representation of distinct authors from Ashkenaz, Spain, and France. To assess the reliability of our findings, we conducted a chi-square test [82]. The chi-square test values χ^2^(5) = 20.69, p = 0.0009, indicates significant differences in resource diversity among the communities.

The rows indicate the percentage of distinct authors from each community cited by a specified community. Each cell’s shading intensity represents the relative indicator’s value.

**Fig 9:**
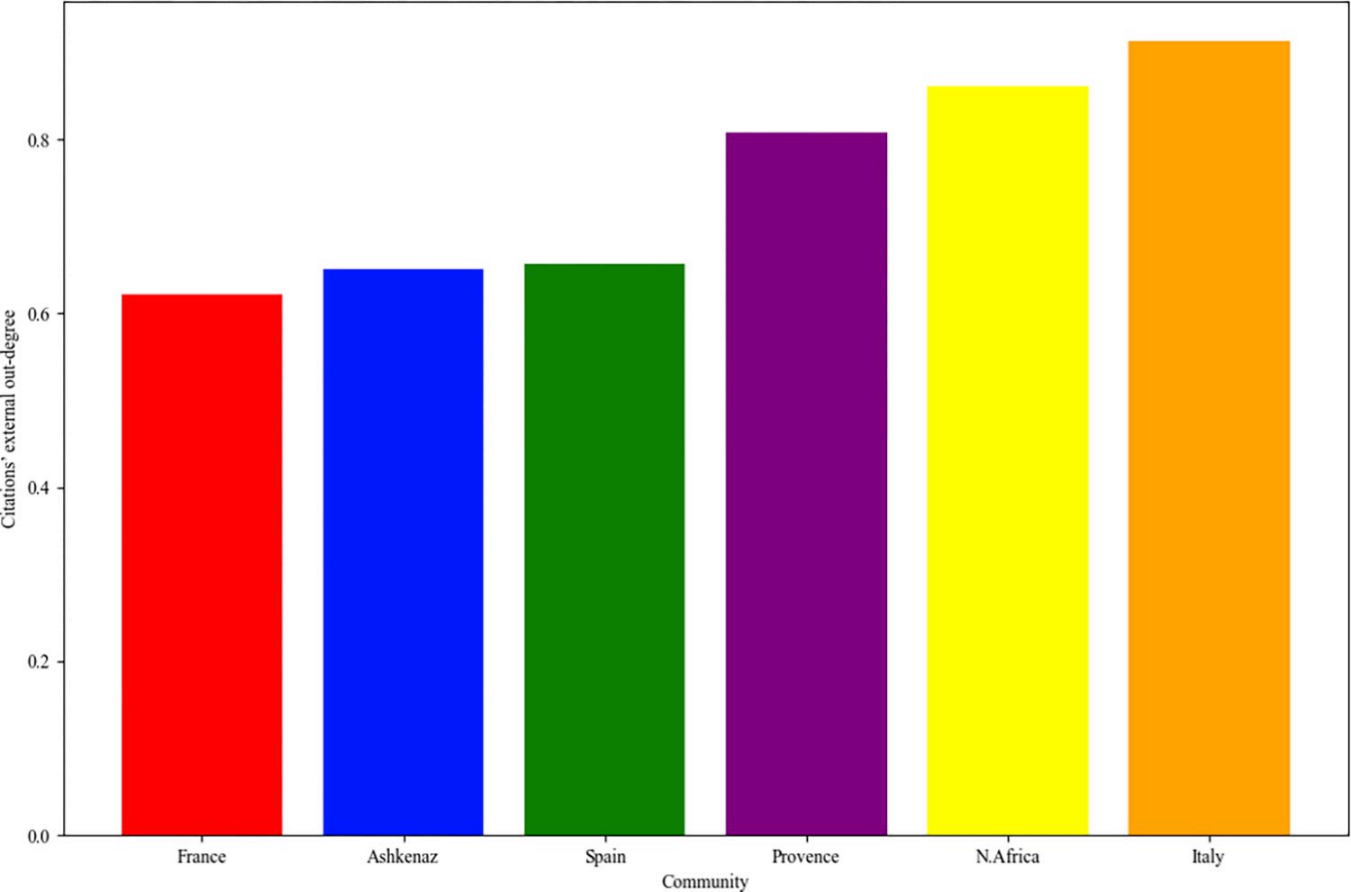
The external citations’ outdegree of each community.

The second indicator assesses each community’s external outdegree. As can be viewed in Fig 9, France shows the least citations of external authors, in contrast to Italy, which presents the highest percentage of external citations.

**Fig 10:**
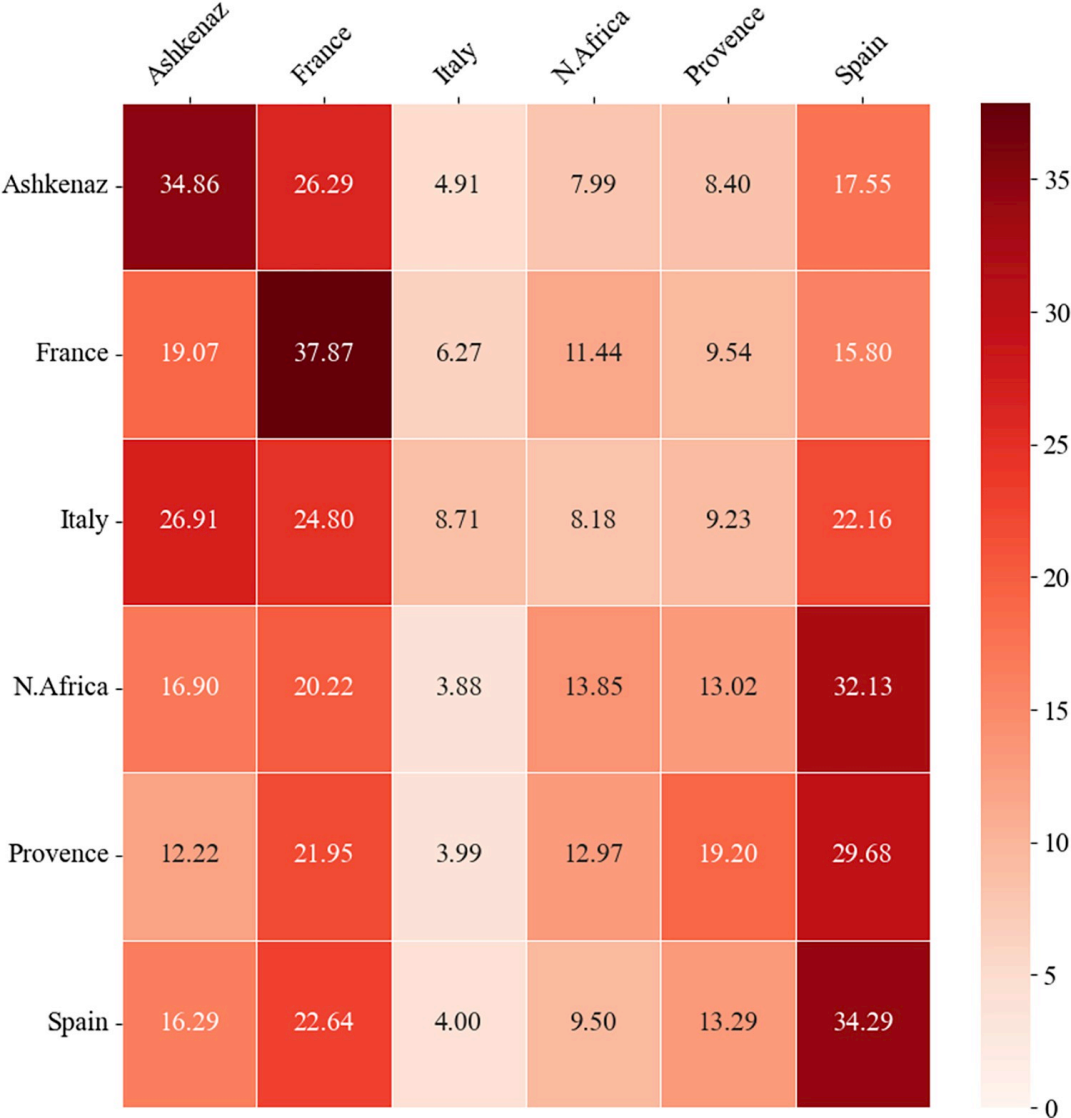
Cross-community outdegree heatmap.

When analyzing the citations based on their target communities, it is observed from Fig 10 that France, Ashkenaz, and Spain most frequently refer to authors from their own communities. In contrast, the three other communities (Provence, Italy and North Africa) demonstrate a higher degree of external citations. The chi-square test demonstrated significant differences among the communities in terms of the external outdegree, χ^2^(5) = 60.50, p- = 9.55e-12.

Cell’s shading intensity in each row represents the percentage of the outgoing citations of the different communities by a given community.

**Fig 11:**
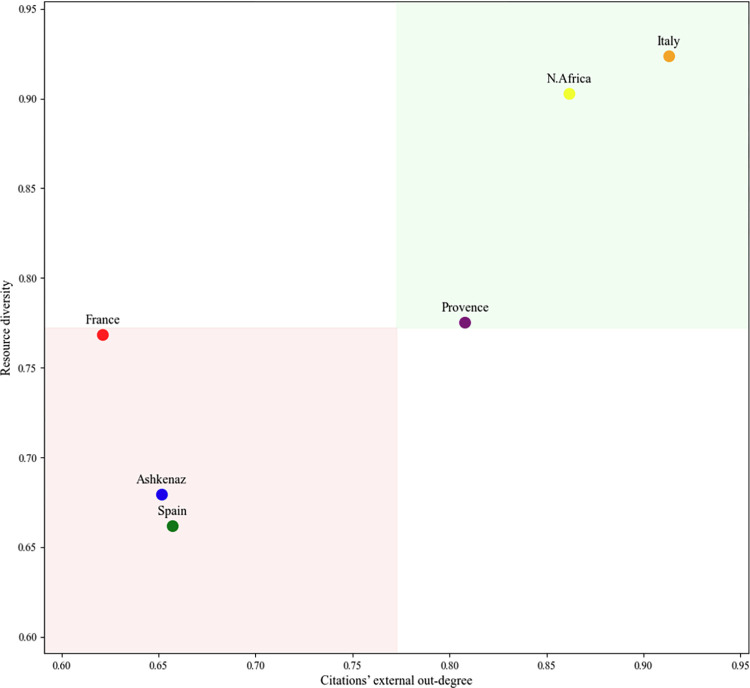
A bi-dimensional visualization illustrates the final plurality level for each community.

Finally, when visualizing the combined plurality score for each community on a bi-dimensional graph (Fig 11), we found that Italy and North Africa demonstrate the highest scores for both indicators. Provence exhibits a significant lower diversity of external resources, but uses them extensively, while France has a similar diversity as Provence, but utilizes these resources to a much lesser extent, suggesting that French authors were aware of authors and Rabbis from other communities, but, ultimately, they did not frequently cite their books compared to their internal author’s citation rates. Spain and Ashkenaz display low levels of both external resource diversity and outdegree.

This visualization represents resource diversity on the Y-axis and external citations’ outdegree on the X-axis, with both axes starting at around 0.6.

## Discussion and conclusion

In this paper, we proposed and implemented a novel analytical framework and methodology to assess viewpoint plurality of historical corpora. The approach is grounded in a large-scale network analysis of citations among individual authors and entire groups or communities with similar characteristics. It scrutinizes the awareness and utilization of resources from counterparts, author groups from different backgrounds who may hold alternative and even conflicting views, assessing the extent to which different authors and groups engage in such practices. Our methodology’s robustness stems from the application of computational network-based methods, facilitating a large-scale analysis of global trends in the entire corpus, as opposed to a restricted manual examination of a specific, limited number of authors and books.

As part of the definition of the analytical framework, we devised a set of quantitative metrics that measure various network features and citation patterns of authors and communities including the standard metrics and novel indicators, such as resource diversity and external citations’ outdegree.

When this method was applied to a large historical Rabbinic literature corpus from the High Middle Ages, comprising over 60 million words and 4110 citations, it was observed that, across all the determined communities, the quantity and diversity of external citations surpassed those of internal citations. Our analysis reveals that Italy, one of the smallest communities in the corpus, demonstrates the greatest degree of plurality, in terms of both the diversity of resources and the external outdegree of citations. This trend is evident at both community and individual author levels. Conversely, Ashkenaz and Spain, the largest communities in the corpus, exhibit the least viewpoint plurality compared to others. This pattern is also observed at the individual author level. The behavior of French authors is more varied; some show a minimal level of viewpoint plurality in terms of resource diversity and external out degree citations, while others demonstrate a significant range in these indicators. As a community, France, in comparison to Ashkenaz and Spain, possesses a greater diversity of resources but a lesser outdegree of external citations. This might suggest that French authors, as a group, are more acquainted with external resources, but prefer citing their own books. In North Africa, a relatively small community, the uniformity in resource diversity is notable. However, the extent of external outdegree citations among authors varies, though, at the community level, North Africa is relatively close to Italy in this regard. Provence, as a community, mirrors France in terms of resource diversity but exhibits a much higher rate of external outdegree citations. The behavior of its authors is relatively consistent across both metrics.

Overall, two conflicting patterns emerge when employing the different network measures. In the initial analysis of the data, utilizing standard network metrics, Spain and Ashkenaz exhibit a greater openness to diverse viewpoints, surpassing others in the total number of outgoing citations and citations of external resources. Conversely, Italy and North Africa seem more closed and univocal. However, a more in-depth investigation, considering viewpoint plurality indicators developed in this study, based on the relative proportions between the external and internal citations, yields a divergent result. Spain and Ashkenaz demonstrate the lowest scores on diversity measures, while Italy and North Africa attain the highest. Consequently, we assert that a relatively higher number of external outgoing citations does not necessarily signify higher levels of plurality and diversity of viewpoints, highlighting one of the notable findings of our study.

Reflecting on the findings pertaining to our specific corpus offers intriguing insights into the concept of plurality in the Rabbinic teachings and intellectual culture. This corpus, designed to delineate practical religious conduct, originated in an era where communication mediums and information dissemination were rudimentary compared to today. Contemporary criticism often targets social media platforms, arguing that they inadvertently trap users within echo chambers that reverberate their own viewpoints [83]. Given this backdrop, one might intuitively anticipate a limited scope of openness and inclusion of diverse viewpoints in the historical religious texts. Contrarily, our results paint a different picture: despite vast geographic and cultural divides, the Medieval Rabbinic texts are full of citations of peers, and especially and quite surprisingly, external cross-community citations that indicate a high level of awareness, plurality, and willingness to acknowledge and contemplate divergent viewpoints.

Practically, the concept of diversity in sources involves recognizing and accepting Halachic rulings and methodologies from various communities. Even if not fully adopted, these rulings and methodologies are considered for their Halachic significance. A notable example is the study and interpretation of the Talmud, which significantly evolved in Ashkenaz and France during the 12th and 13th centuries. Initially focused on localized study and interpretation of specific issues, this approach developed into a broader methodology that sought to align and integrate matters across different tracts, resulting in a more cohesive interpretation [12]. This methodology reached Spain in the 13th century through rabbis like Ramban and the Beit Midrash he established [12]. Provence serves as a good example of a region that adopted methodologies and texts from both France and Spain. This blend led to the emergence of two distinct factions within Provence: one inclined towards French influences and the other towards Spanish. This convergence sparked a dynamic and diverse body of Halachic literature [84]. While our research did not focus on these methodological changes, our findings regarding the impact of France on other communities and the diversity exposed by Provence could support and contribute to research on these methodological changes investigated by classical Jewish research literature.

Another aspect that may influence our results is the presence of "classic" authors who are cited by all communities, potentially introducing bias. However, in our study, almost all "classical" authors universally accepted across different communities during our research period were the Geonim, who lived up to the 11^th^ century [85]. Since this period is not included in our research, we excluded these early authors. Even Rashi, the famous French commentator who gained widespread influence during the High Middle Ages, was not recognized by eastern countries until the 13th century [86]. Thus, in our corpus, "classical" authors and resources do not introduce bias into our results. Additionally, traditional Jewish studies research literature considers France the greatest influencer in the Middle Ages (particularly in the 12^th^ and 13^th^ centuries) due to the work of the Tosafists [12]. They expanded and refined the interpretation of the Talmud beyond what Rashi had provided [67].

Generally, in this study, we measure the diversity of outgoing citations of authors and communities rather than just the absolute number of incoming citations, that are not expected to be biased by a few “classical” authors with high numbers of incoming citations. Berlin [87] argues that the inclination towards pluralism and diversity is a relatively recent evolution, emerging around the 18th century. Classical thought held that every query has a singular, definitive answer that is not only attainable but also harmonious with other established truths. Nevertheless, Sagi [88] challenges this idea by demonstrating that various Rabbinic authors and books displayed more nuanced and complex perspectives towards the notion of seeking truth and the acceptance of diverse viewpoints as legitimate. Our results support this hypothesis.

**Fig 12:**
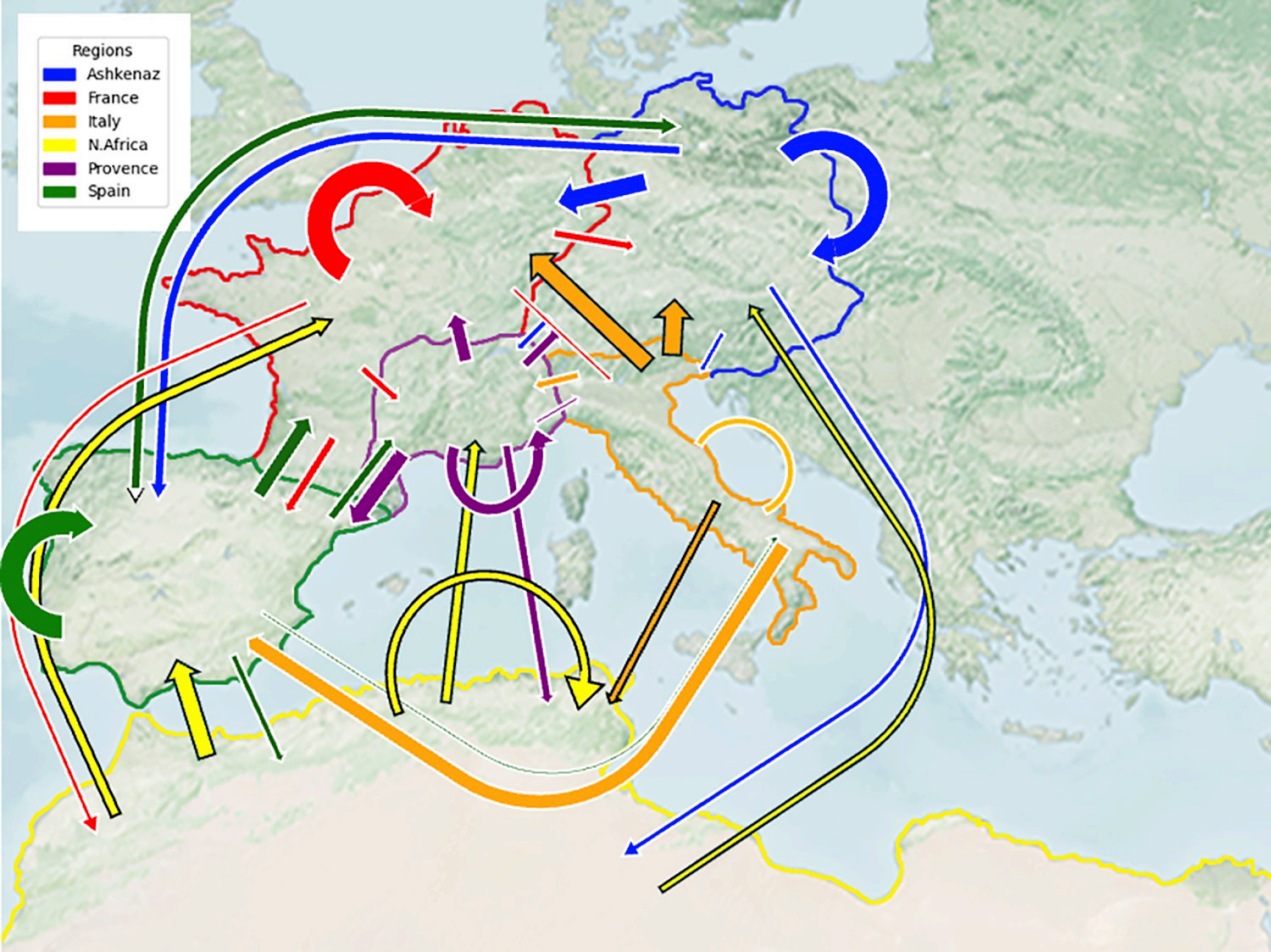
A map displays arrows indicating cross-community references among major Jewish communities.

Historians and scholars specializing in Rabbinic literature possess the expertise to provide interpretation and conduct close-reading analysis of the phenomena identified in this study. The reasons for the identified phenomena could be multifaceted, including the different historical periods and geographic location in which the authors lived, community sizes, intra-community author relationships, the degree of students’ adherence to their teachers, authors’ and communities’ migration and major historical events that influenced the Jewish communities [64,89]. Moreover, the citation network, mapping the inter-community relationships (Fig 12), may offer valuable insights into the cultural ties and influences among dispersed Jewish communities worldwide. Notably, these communities, despite significant geographic and temporal distances and the absence of modern information and communication technologies and transportation, demonstrated awareness and inclusiveness of each other’s works.

These communities are located in six regions: North Africa, Spain, Provence, Italy, France, and Ashkenaz (mostly modern-day Germany), with the arrow width representing the volume of outdegree citations. The image was sourced from NASA’s Earth Observatory website (https://earthobservatory.nasa.gov/map#3/21.78/-10.20) and has been modified for illustrative purposes only.

In addition to its methodological and scientific contributions, the study holds practical societal value. The network and metadata ontology of the corpus have been uploaded to Wikidata, the largest open linked data initiative, making this information freely accessible to researchers and the general public.

This research is subject to various limitations. Handling historical texts presents a significant challenge for automated text processing and citation extraction, given the diverse cultural backgrounds, locations, and periods of the numerous individuals who authored them, utilizing distinct writing styles and vocabulary. Consequently, the constructed network might contain errors and may not capture all the citations in the corpus. Nevertheless, we assert that the general trends unveiled through statistical analysis of the network based on the devised quantitative metrics remain valid. Furthermore, as the authors within the examined communities lived in different periods, a diachronic analysis of the network could uncover additional discrepancies, offering valuable insights into the obtained results. Another promising avenue for future research involves a deep semantic analysis of citation contexts to identify sentiment towards the cited resources, enhancing the construction of an accurate and refined multi-viewpoint map of the authors’ and communities’ relationships in the corpus. Another type of citation extraction that could be explored in a future study involves identifying textual quotations in books that lack explicit attribution, tracing their sources, and integrating this information into the existing citation network. This approach will offer a clearer understanding of the resources utilized by authors and enrich our research data. Finally, the established method can be adapted for exploring similar inquiries within other textual collections, such as various legal and literary corpora.
